# The expression profile of genes associated with behavior, stress, and adult neurogenesis along the hippocampal dorsoventral axis in tame and aggressive foxes

**DOI:** 10.18699/VJGB-23-76

**Published:** 2023-10

**Authors:** Yu.V. Alexandrovich, E.V. Antonov, S.G. Shikhevich, A.V. Kharlamova, L.V. Meister, Y.V. Makovka, D.V. Shepeleva, R.G. Gulevich, Yu.E. Herbeck

**Affiliations:** Institute of Cytology and Genetics of the Siberian Branch of the Russian Academy of Sciences, Novosibirsk, Russia; Institute of Cytology and Genetics of the Siberian Branch of the Russian Academy of Sciences, Novosibirsk, Russia Sirius University of Science and Technology, Scientific Center for Translational Medicine, Sochi, Russia; Institute of Cytology and Genetics of the Siberian Branch of the Russian Academy of Sciences, Novosibirsk, Russia; Institute of Cytology and Genetics of the Siberian Branch of the Russian Academy of Sciences, Novosibirsk, Russia; Institute of Cytology and Genetics of the Siberian Branch of the Russian Academy of Sciences, Novosibirsk, Russia; Institute of Cytology and Genetics of the Siberian Branch of the Russian Academy of Sciences, Novosibirsk, Russia; Institute of Cytology and Genetics of the Siberian Branch of the Russian Academy of Sciences, Novosibirsk, Russia; Institute of Cytology and Genetics of the Siberian Branch of the Russian Academy of Sciences, Novosibirsk, Russia; Institute of Cytology and Genetics of the Siberian Branch of the Russian Academy of Sciences, Novosibirsk, Russia Koret School of Veterinary Medicine, The Robert H. Smith Faculty of Agriculture, Food and Environment, The Hebrew University of Jerusalem, Rehovot, Israel

**Keywords:** tame behavior, aggression, domestication, silver fox, cortisol, ручное поведение, агрессия, доместикация, серебристо-черные лисицы, кортизол

## Abstract

The hippocampus plays the key role in stress response regulation, and stress response appears to be weakened in domesticated animals compared to their wild relatives. The hippocampus is functionally heterogeneous along its dorsoventral axis, with its ventral compartment being more closely involved in stress regulation. An earlier series of experiments was conducted with a unique breeding model of animal domestication, the farm silver fox (Vulpes vulpes), which included tame, aggressive, and unselected animals. A decrease in many indices of the hypothalamic–pituitary–adrenal activity was observed in tame animals. Also, adult hippocampal neurogenesis was more intense in tame foxes, and this fact may relate to reduced stress levels in this experimental population of foxes. Nevertheless, the molecular mechanisms responsible for the reduced stress response in tame animals remain obscure. In this study, serum cortisol levels and the mRNA levels of 13 genes in the dorsal and ventral hippocampus have been measured and compared in tame, aggressive, and unselected foxes. At the current stage of domestication, stress-induced cortisol levels in tame, aggressive, and unselected animals differ significantly from each other: tame foxes show the lowest levels, and aggressive ones, the highest. Twelve genes tested demonstrate significant gene expression differences between the dorsal and ventral hippocampi. These differences are mainly consistent with those found in rodents and humans. In tame foxes, significantly elevated mRNA levels were recorded for several genes: CYP26B1 for cytochrome P450 26B1 and ADRA1A for α1A adrenergic receptor in the dorsal hippocampus, whereas the level of NR3C2 mRNA for mineralocorticoid receptor was higher in the ventral. It is presumed that these genes constitute an important part of the mechanism reducing stress induced by contacts with humans and contribute to linking stress regulation with adult neurogenesis in tame foxes and domesticated animals in general.

## Abstract (ru)

Гиппокамп является ключевой структурой в регуляции стресс-ответа, который, по-видимому, снижен у домашних животных по сравнению с их дикими сородичами. Известно, что гиппокамп функционально неоднороден вдоль дорзовентральной оси, и в регуляции стресса в большей мере участвует вентральная часть. В серии экспериментов на уникальной селекционной модели одомашнивания животных – серебристо-черной лисице (Vulpes vulpes), включающей ручных, агрессивных и неселекционированных животных, ранее было показано снижение активности гипоталамо-гипофизарно-надпочечниковой системы во многих звеньях. Кроме того, известно, что уровень нейрогенеза в гиппокампе повышен у взрослых ручных лисиц, что может быть взаимосвязано со снижением уровня стресса. Тем не менее молекулярно-генетические механизмы снижения стресс-ответа у доместицированных животных по-прежнему не ясны. В настоящей работе выполнено сравнение мРНК 13 генов в дорзальном и вентральном гиппокампе и проведен анализ кортизола в крови у ручных, агрессивных и неселекционированных лисиц. Установлено, что на данном этапе доместикации стресс-индуцированный уровень кортизола у ручных, агрессивных и неселекционированных животных достоверно отличается друг от друга, причем у ручных животных он самый низкий, а у агрессивных – самый высокий. Выявлены достоверные различия в экспрессии 12 генов между дорзальной и вентральной частями гиппокампа, что в большинстве случаев соответствует аналогичным различиям, найденным у грызунов и человека. У ручных лисиц обнаружен достоверно повышенный уровень в дорзальном гиппокампе мРНК генов цитохрома P450 26B1 (CYP26B1) и адренергического рецептора α1A (ADRA1A), а в вентральном гиппокампе - мРНК гена минералокортикоидного рецептора (NR3C2). Эти гены могут быть важной частью механизма снижения стресса по отношению к человеку и взаимосвязи регуляции стресса и нейрогенеза у взрослых ручных лисиц в частности и доместицированных животных вообще.

## Introduction

The hippocampus is an important brain region involved in the regulation of stress response, learning, spatial memory, social recognition, and memory consolidation. Hippocampus sizes in mammals and birds and adult neurogenesis in mammals are likely to correlate with the capacity for spatial orientation and memory. Also, changes in hippocampus morphology may be associated with adaptive evolution (Jacobs et al., 1990; Jacobs, Spencer, 1994; Rehkämper et al., 2008; Croston et al., 2015; Sonnenberg et al., 2019). However, other scientists presume that neurogenesis rate and hippocampus size, albeit adaptive, are not related to memory or spatial orientation (Lipp, 2017). The 15-fold increase in neurogenesis rate in adult red foxes as compared to dogs (Amrein, Slomianka, 2010) may be related to spatial memorization, typical of foxes hoarding food (Sklepkovych, Montevecchi, 1996). It is known that the CA2 region takes part in social recognition memory (Tzakis, Holahan, 2019). The CA1 and CA3 volumes in primates appear to be related to social and environmental signals, such as group and home range sizes (Todorov et al., 2019).

The hippocampus is among the key elements of the central regulation of the hypothalamic–pituitary–adrenal (HPA) axis. It is known to be functionally and structurally heterogeneous along its dorsoventral axis. It is believed that the regulation of HPA axis action and stress and emotional responses are governed primarily by the ventral hippocampus, and cognitive functions, by the dorsal (O’Leary, Cryan, 2014; Gulyaeva, 2019). This specialization may be due to the locations of these hippocampus compartments, and, correspondingly, the greater number of dorsal hippocampus projections to the crust and of ventral hippocampus projections to regions belonging to the limbic system (O’Leary, Cryan, 2014).

Along with the lateral ventricle subependymal zone, the hippocampus is a region where neurogenesis occurs constantly, even in adulthood (Ming, Song, 2011). The neurogenesis rate is reduced by stress in most cases, and, vice versa, high neurogenesis rate mitigates the effect of stress on the hippocampus (Levone et al., 2015). Thus, stress and the hippocampus functional response exert reciprocal actions. Apparently, the effect of stress on neurogenesis varies along the dorsoventral axis (O’Leary, Cryan, 2014). This inference may explain the fact that the hippocampus neurogenesis rate in tame foxes, whose stress response is much weaker, is higher than in unselected ones, and the most pronounced differences are recorded in the ventral and intermediate compartments of the hippocampi (Huang et al., 2015). Studies on dogs have shown that differences between the dorsal and ventral hippocampi are at the same level despite considerable variations in the overall neurogenesis rate among individuals (Lowe et al., 2015).

Experiments with hippocampus samples from rats, mice, and humans reveal differences in gene expression between the dorsal and ventral hippocampi. They are likely to reflect the functional and structural heterogeneity along the axis (Cembrowski et al., 2016; Lee et al., 2017; Floriou-Servou et al., 2018; Vogel et al., 2020). However, the expression profiles of these genes along the hippocampus dorsoventral axis have not been studied in other taxa, in spite of their functional and structural features

These data motivated us to investigate gene expression variation along the dorsoventral axis and seek molecular mechanisms linking neurogenesis and stress by quantitation of mRNAs of 13 genes in the dorsal and ventral hippocampi of silver foxes. This species serves as a model of animal domestication. Its “tame” and “aggressive” populations had been raised by long-term selection for friendly or aggressive attitude to humans, respectively, and foxes not subjected to targeted selection for behavior served as control.

These populations differ significantly in many links of their glucocorticoid stress response and HPA axis activity, which seems to be a common feature of domestic animals (Belyaev, 1979; Price, 2000; Trut et al., 2004, 2009), and in adult neurogenesis in the hippocampus (Huang et al., 2015). Genes of the retinoic acid pathway, associated with neurogenesis activity, which are located in regions presumably affected by the selection, have been found in genome-wide analysis of the tame foxes (Kukekova et al., 2018; Trut et al., 2021). Also, it has been found that the amount of mRNA of one of these genes, CYP26B1, in the dorsal hippocampus of tame foxes differs from aggressive ones (unpublished results). This may be one of the mechanisms altering adult neurogenesis in foxes, which can also modulate stress level, learning, memory, and social behavior. For all that, nothing is known about changes in CYP26B1 mRNA levels in the ventral hippocampi of foxes with contrasting behaviors, although its elevated expression characterizes the dorsal hippocampus in mice, rats, and humans

We also measured the dorsal and ventral hippocampus levels of mRNAs of other genes related to neurogenesis, stress, or behavior whose expression is known to vary significantly along the dorsoventral axis in mice, rats, and humans (Vogel et al., 2020) (see Table 1). The genes associated with HPA axis regulation include NR3C1, NR3C2, and HSD11B1 for glucocorticoid receptors 1 and 2 and hydroxysteroid 11β-dehydrogenase 1, respectively (de Kloet et al., 2016). NR2F2 is one of the most reliable markers of the position along the dorsoventral hippocampus axis, and it supposedly acts as a mediator of the transcription activity induced by receptors of glucocorticoids and retinoic acid. This action is likely to be associated with relationships between stress and neurogenesis (de Martino et al., 2004; Vogel et al., 2020). The ADRA1A gene for the α1A adrenergic receptor is presumed to act in the regulation of behavior and neurogenesis (Doze et al., 2011; Vogel et al., 2020). KCND2, KCND3, CADM2, and CPNE2 are associated with K+- and Ca2+-dependent synaptic and glutamatergic transmissions (Corradini et al., 2014; Truvé et al., 2020; Haddjeri-Hopkins et al., 2021; Xiao et al., 2021), which are likely to play the key role in domestication-driven behavior changes (O’Rourke, Boeckx, 2020; Trut et al., 2021). The expression levels of TRHR, on the one hand, and LCT, NTS, on the other hand, have been used as markers of the ventral and dorsal hippocampi, respectively (Cembrowski et al., 2016; Lee et al., 2017).

Earlier data on the glucocorticoid-mediated stress response in foxes demand re-research at the present phase of selection. After over sixty generations of the selection of Norway rats according to approximately the same criteria as foxes as an alternative experimental domestication model, differences between tame and aggressive animals in the glucocorticoid-mediated stress response vanished. This might result from the adaptation of rats selected to aggression toward humans (Prasolova et al., 2014). Besides, previous studies employed nonsocial restriction stress (Trut et al., 2004, 2009). This procedure is less adequate than the use of social stress in studies of animal–animal and animal– human contacts. It has been shown that restriction stress in rats selected for anxiety-like behavior induces a stronger corticosterone-mediated response than in animals selected for low levels of such behavior, whereas social stress in the resident–intruder test shows quite the opposite (Veenema, Neumann, 2007). Here we studied the stress response to a combined treatment: manual fixation of foxes by a social subject, human. Manual fixation for 15 min was a stress factor for all the three fox populations, because foxes had never been picked in arms during selection and they had had an opportunity to avoid close contacts with humans.

## Materials and methods

Experimental animals. Experiments were conducted with three experimental populations of silver foxes (Vulpes vulpes): tame, aggressive, and unselected. The first two populations had been selected for friendly and aggressivefearful reactions to humans, respectively, at the Shared Access Center for Gene Pools of Fur and Farm Animals, Institute of Cytology and Genetics, Novosibirsk, for over 60 years (Belyaev, 1979; Trut et al., 2004, 2009).

Blood was sampled from the vena saphena of 6–7-monthold males prior to experimental stress (manual fixation for 15 min) and immediately after it. Naïve (having experienced no experimental stress) 7–8-month old males were euthanized by injections of 5 % sodium thiopental. Fragments of the dorsal and ventral hippocampi were sampled. All samples were stored at –70 °C. Experimental protocols followed the Guidelines for Accommodation and Care of Laboratory Animals, Species-specific Provisions for Laboratory Predatory Mammals, GOST 33217-2014, and Directive 2010/6106/ EU for the Protection of Laboratory Animals

Chromatography. Blood serum cortisol was assayed by high-performance liquid chromatography with an Agilent 1200 Series LC chromatograph equipped with a diodearray detector and a ZORBAX C18 2 × 150 mm × 5 μm column, as in previous studies (Ovchinnikov et al., 2018). Samples were concentrated by liquid extraction with 1,2-dichloroethane. Elution was done with 30 % aqueous acetonitrile at the rate 1 mL/min. Absorption was measured at λ = 246 nm. Concentrations were calculated against dexamethasone as an internal standard.

Total RNA isolation and real-time PCR. RNA was isolated with TRI Reagent (Molecular Research Center, Inc.) according to manufacturer’s recommendations, as in (Ovchinnikov et al., 2018). Absorption values were measured with a NanoPhotometer N50 (Implen, Germany). For RNA purity assessment, A260/A280 and A260/A230 ratios were calculated. Genomic DNA was removed from RNA samples with a DNase I, RNase-free kit (Thermo Fisher Scientific, Lithuania). cDNA was synthesized in a 20 μL volume with 0.2 μg of DNA-free RNA and a Maxima First Strand cDNA Synthesis Kit for RT-qPCR (Thermo Fisher Scientific, Lithuania).

Primers for the genes studied were designed using the online web resource Primer-BLAST (Ye et al., 2012). The sequences are shown in Table 1.

**Table 1.**
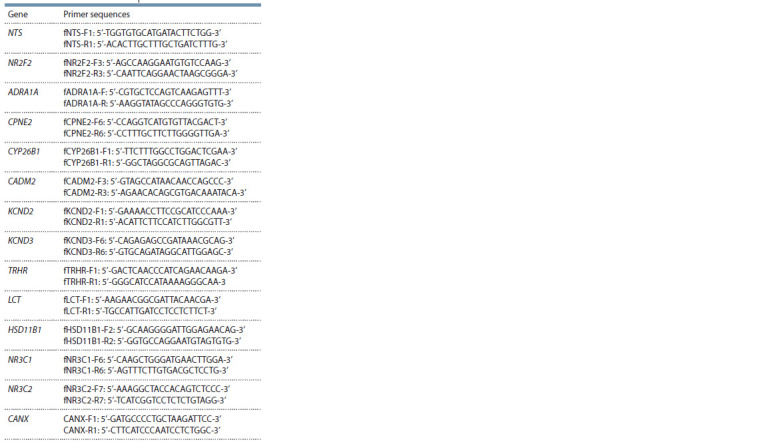
Primers for real-time PCR

Real-time PCR was conducted in a Roche LightCycler 96 Real-Time PCR System (Roche Diagnostics, Switzerland). The reaction volume 20 μL contained 4 μL of twentyfold diluted cDNA, 0.3 μL of primers (10 pmol/μL), 7.4 μL of Milli-Q H2O, 8 μL of 2.5× reaction mix for Real-Time PCR, and SYBR Green I dye (Mfr. Part No. M-427, Syntol, Russia). Each reaction was performed in two technical replications

The results were processed by the modified ΔΔCt method (Livak, Schmittgen, 2001) implemented in GenEx ver.6 software (Multi-D, Sweden). This method allows the reaction efficacy to be estimated. The CANX gene for calnexin was used as reference, because its expression is high, little variable among individual foxes, and uniform in the dorsal and ventral hippocampi, as confirmed by analysis with NormFinder software (Andersen et al., 2004). The mean expression of each gene was taken to be an arbitrary unit for the evaluation of relative expression. An additional external reference sample was present in all plates for proper comparison of the results obtained in different plates.

Statistical evaluation. The statistical significance of hormone assays in the experimental groups was assessed by repeated measures factor analysis followed by post hoc Fisher’s LSD test. The increase in cortisol level was assessed by Student’s t test.

Real-time PCR results were compared by the Kruskal– Wallis test. Pairwise comparisons were done by the Mann– Whitney test. We applied nonparametric criteria, because the samples did not conform to the Gaussian distribution according to the Kolmogorov–Smirnov test.

Use was made of software packages Statistics 10 (Stat- Soft, United States) and GenEx ver.6 (Multi-D, Sweden). All differences were considered significant at p < 0.05. The results are shown in figures as mean ± SEM.

## Results

Blood serum cortisol in response to stress

**Fig. 1.**
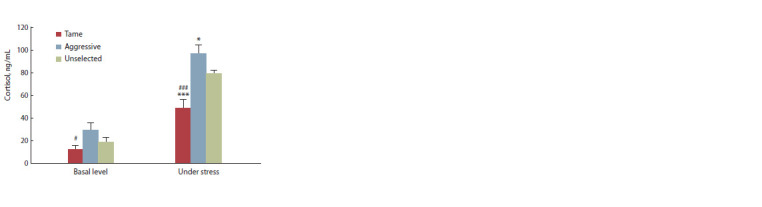
The basal and stress-induced blood serum cortisol levels in tame, aggressive, and unselected foxes (n = 11 in each group). * p < 0.05, *** p <0.001 in comparison with unselected foxes. # p < 0.05, ### p < 0.001 in comparison with aggressive foxes.

Repeated measures factor analysis reveals the effects of genotype (F2.28 = 9.62, p < 0.001) and stress (F2.28 = 179.72, p < 0.001) on blood serum cortisol. The interaction of the genotype and stress factors was also significant (F2.28 = 9.36, p < 0.01). The basal serum cortisol level in tame foxes was lower than in aggressive (Fig. 1, p < 0.05) but did not differ significantly from unselected ones. The increase in serum cortisol level was statistically significant in all genotypes, but it was less pronounced in tame foxes than in aggressive or unselected. Thus, the cortisol level under stress in aggressive and unselected foxes exceeded the value in tame ones ( p 0.001, see Fig. 1) and was higher in aggressive foxes than in unselected ( p < 0.05, see Fig. 1).

Levels of mRNAs in the dorsal and ventral hippocampi of foxes

**Fig. 2.**
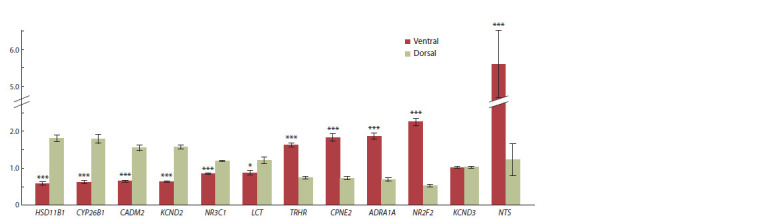
Differences between the dorsal and ventral hippocampi in relative mRNA levels (n = 18 in each group). * p <0.05, *** p < 0.001 as compared to the dorsal hippocampus.

Quantitative real-time PCR revealed in the dorsal hippocampus significantly higher levels of the genes HSD11B1, CYP26B1, CADM2, KCND2, NR3C1, LCT, and NR3C2 and in the ventral, TRHR, CPNE2, ADRA1A, and NR2F2, whereas KCND3 showed no difference (Fig. 2, Table 2).

**Table 2.**
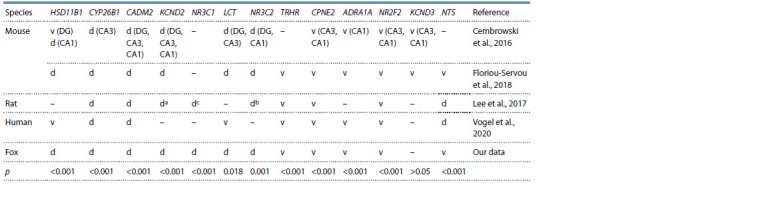
Differences between the dorsal and ventral hippocampi in gene expression in mammals Notе. CA1, CA3, and DG (dentate gyrus) are hippocampus regions; d, mRNA level is higher in the dorsal hippocampus; v, mRNA level is higher in the ventral hippocampus; –, no difference or not known. p values are indicated according to the Mann–Whitney test. a 28 and 45 days (growing animals); b 28 days; c rat Nr3c1 mRNA according to Kvichansky et al. (2017).

NTS mRNA levels showed a broad variation among hippocampi of individuals, but the expression in the ventral compartment was always higher (see Fig. 2, Table 2), and so was the neurogenesis rate in a study on dogs reported by Lowe et al. (2015).

Levels of mRNAs in the hippocampi of tame, aggressive, and unselected foxes

**Fig. 3.**
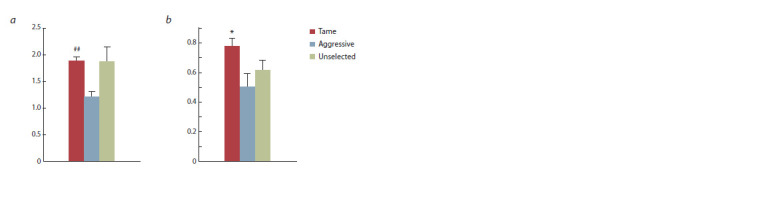
Relative amounts of CYP26B1 (a) and ADRA1A (b) mRNA in the dorsal hippocampi of tame (n = 7), aggressive (n = 6), and unselected (n = 6) foxes. ## p <0.01 as compared to aggressive foxes; * p < 0.05 as compared to aggressive and unselected foxes.

**Fig. 4.**
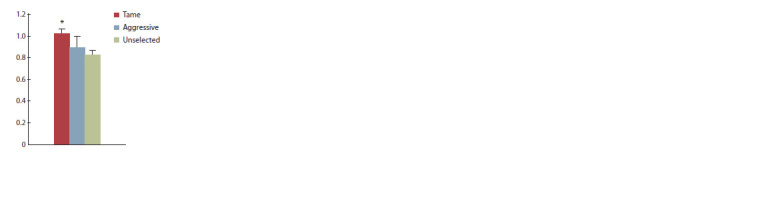
Relative amounts of NR3C2 mRNA in the ventral hippocampi of tame (n = 7), aggressive (n = 6), and unselected (n = 6) foxes. * p < 0.05 as compared to unselected foxes.

We compared the levels of mRNAs indicated in Table 2 in tame, aggressive, and unselected fox groups by the Kruskal– Wallis test and revealed effects of genotype on mRNAs of the genes (1) CYP26B1 (H (2, n = 19) = 8.89; p = 0.02) and ADRA1A (H (2, n = 19) = 7.81; p = 0.02) in the dorsal compartment and (2) NR3C2 for a mineralocorticoid receptor (H (2, n = 19) = 7.07; р = 0.03) in the ventral compartment. Tame foxes showed a significant increase in the CYP26B1 mRNA as compared to aggressive animals ( p = 0.003) (Fig. 3, a) and in ADRA1A as compared to both aggressive ( p = 0.027) and unselected ( p = 0.038) (see Fig. 3, b). Also, the NR3C2 mRNA level in the ventral hippocampus of tame foxes was significantly higher than in unselected ones ( p = 0.011) (Fig. 4).

## Discussion

The presented data point to significant differences between the dorsal and ventral hippocampi in the levels of some mRNAs in foxes, as well as in other species: rats, mice, and humans (Cembrowski et al., 2016; Lee et al., 2017; Floriou-Servou et al., 2018; Vogel et al., 2020). Our results are by and large consistent with literature data on rats and mice (see Table 2). Recent studies of human gene expression (Allen Brain Atlas) demonstrate variation in the expression of about 5,000 genes along the hippocampus dorsoventral axis (Vogel et al., 2020). Some of these genes show linear or near-linear variation; thus, they can be regarded as axial position markers. Others demonstrate nonlinear expression profiles.

The NR2F2 gene for transcription factor COUP-TFII is one of the markers most precisely indicating axial position. The amount of its mRNA in the ventral hippocampus is much greater than in the dorsal in all studies on rats, mice, and humans and in our study on foxes. Its expression in the adult hippocampus is confined mainly to GABAergic and glutamatergic neurons. The density of GABAergic neurons increases along the dorsoventral axis (Jinno, Kosaka, 2010), and so does NR2F2 expression. Nevertheless, its expression in the dorsal hippocampus is confined to GABAergic neurons, which is indicative of their high density in the dorsal hippocampus as well (Fuentealba et al., 2010). The different functions of hippocampus compartments may be related to neuron distribution along the dorsoventral axis. It is known that the expression of acetylcholine receptor α7 (CHRNA7) in the hippocampus, also confined to GABAergic neurons, is involved in aggression regulation (Lewis et al., 2018). However, we found no difference in NR2F2 expression amongst the behavior groups; hence, aggression is controlled by other mechanisms.

Different species demonstrate inverse ratios between mRNA levels of some genes in the dorsal and ventral hippocampus compartments or no variation at all (see Table 2). For example, in our study such genes included KCND3 and NTS. These differences can be explained by nonlinear expression profiles along the dorsoventral axis and putative sampling from nonidentical hippocampus sites in different experiments. In other cases, the species-specific expression of some genes may be related to morphological and functional features of the hippocampus itself. For instance, it is known that hippocampal neurogenesis in foxes considerably surpasses that in many mammals (Amrein, Slomianka, 2010). Morphological and functional features of various species may also stem from their ecology, in particular, spatial behavior in hoarding food (Jacobs et al., 1990; Jacobs, Spencer, 1994; Rehkämper et al., 2008; Amrein, 2015; Croston et al., 2015; Lipp, 2017; Sonnenberg et al., 2019). It is presumable that the 15-fold hippocampal neurogenesis in red foxes as compared to dogs (Amrein, Slomianka, 2010) is related to this characteristic behavior (Sklepkovych, Montevecchi, 1996).

As the entire hippocampus and, especially, its ventral compartment, is the key region in stress response regulation, we investigated the glucocorticoid-mediated response of foxes at the present stage of selection: tame, aggressive, and unselected. The necessity of studying stress response at different stages of selection has been shown on another model, tame and aggressive Norway rats, which show no significant differences in the glucocorticoid-mediated response at the current stage (Prasolova et al., 2014). The detected significant differences amongst tame, aggressive, and unselected animals are consistent with earlier data (Trut et al., 2009). Thus, we can see that the restraint and combined restraint–emotional stresses induce similar differences in the cortisol-induced stress response in the experimental foxes, and these differences persist at the current stage of selection. However, the unselected animals showed an intermediate level of the glucocorticoid stress response between the tame and aggressive foxes. These discrepancies may be related to both the elevated stress response in the selection for aggressiveness and the unintentional selection of “unselected” foxes towards adaptation to coexistence with humans. Differences can also stem from different experiment designs (restraint stress with limited space in a shed vs. manual fixation) and measurement protocols (radioimmunoassay vs. HPLC).

It is likely that the weak response to different stress types in tame foxes is the main cause of high adult neurogenesis rate in the hippocampus, as shown in many studies on other species (Levone et al., 2015). Therefore, in search for molecular mechanisms modulating neurogenesis we first considered the levels of mRNAs for glucocorticoid (NR3C1, GR) and mineralocorticoid (NR3C2, MR) receptors in the hippocampus. However, we found no differences in the levels of NR3C1 mRNA amongst animals of different behavior genotypes in neither dorsal nor ventral hippocampus, although some research teams believed that these genes were important in domestication (Oskina et al., 2008; Pörtl, Jung, 2017). It is known that neuron progenitors in the subventricular zone express NR3C1 but not NR3C2 (Garcia et al., 2004). Apparently, NR3C1 activation in these cells is the direct way by which glucocorticoids affect neurogenesis (Saaltink, Vreugdenhil, 2014). It is conceivable that NR3C1 expression in this subpopulation of hippocampus cells varies amongst foxes differing in behavior.

In contrast, the ventral hippocampi of tame foxes contained more NR3C2 mRNA than those of aggressive animals. It is likely that the mRNA level in the ventral hippocampi of unselected foxes is intermediate between tame and aggressive, but this difference is below the limits of qPCR accuracy. However, we may expect that in studies of individual splice variants (Three are known in rodents: α, β, and γ.) differences amongst groups in the expression of a particular splice variant will be more pronounced. It has been shown that their expression in cellular stress in a primary culture of cortex cells varies irregularly (Kang et al., 2009). Here we analyze only the total pool of NR3C2 mRNA, because the fox genome had not been annotated in sufficient detail. It is known that NR3C2 levels in various types of hippocampus cells are different (Le Menuet, Lombès, 2014). Note that the difference was found just in the ventral hippocampus, whose contribution to stress response and emotion regulation is thought to be greater than that of the dorsal (Gulyaeva, 2019).

Studies on rodents demonstrate that elevated MR amount is associated with lower anxiety and active strategy of coping with stress. In females, it is also associated with weaker stress response (Lai et al., 2007; Rozeboom et al., 2007; Kanatsou et al., 2015; de Kloet et al., 2016). In addition, postmortem studies of humans show that depression lowers MR in the frontal (corresponding to rodent ventral) but not occipital (dorsal) hippocampus compartments, with no variation in GR (Medina et al., 2013). Changes in MR expression in rats under stress alter synaptic plasticity in the ventral but not dorsal hippocampus (Maggio, Segal, 2009; O’Leary, Cryan, 2014). It is likely that NR3C2 expression in neuroglia can increase neurogenesis in the ventral hippocampus under acute stress (Le Menuet, Lombès, 2014; O’Leary, Cryan, 2014). Also, progenitor cell proliferation is lowered in mice with NR3C2 knockout (Gass et al., 2000), whereas enhanced NR3C2 expression in the forebrain accelerates progenitor proliferation and increases the population of young neurons in the dentate gyrus (Kanatsou et al., 2017). On the other hand, MR signaling in the hippocampus is involved in the regulation of the start and amplitude of stress response (Ratka et al., 1989; Harris et al., 2013; de Kloet et al., 2016), and stress initiation increases the MR level in the hippocampus (Veenema et al., 2003; de Kloet et al., 2016). The opposite effects of MR and their agonists and antagonists found in various studies may be related to different MR levels in animals at the start of the experiment (de Kloet et al., 2016). We conjecture that the detected high level of mRNA for MR (but not for GR) in the ventral hippocampus of tame foxes is one of the mechanisms that mitigate stress and anxiety in experimental domestication and, probably, indirectly enhance neurogenesis in this compartment. Further studies should be dedicated to the expression of splice variants of MR and their distribution in the hippocampus.

Foxes of different behavior genotypes differed in the contents of СYP26B1 mRNA in the dorsal hippocampus and ADRA1A in the ventral one. As mentioned above, these contents varied along the dorsoventral axis of the hippocampus. The variation in mRNA contents found in the analysis of few samples from the dorsal hippocampi of tame and aggressive foxes by the RNAseq method (unpublished data) also points to the necessity of a comprehensive study of CYP26B1 expression in hippocampus regions of the three fox populations. The CYP26B1 gene encodes an enzyme of the cytochrome P450 superfamily. This enzyme catalyzes the degradation of all-trans retinoic acid (atRA), a vitamin A derivative. Changes in CYP26B1 expression may be associated with different atRA concentrations in the hippocampi of tame and aggressive foxes.

It is known that atRA affects neurogenesis, but its effect looks graphically as an inverted U curve. All-trans retinoic acid deficiency reduces neuron differentiation, whereas higher concentrations enhance neurogenesis by stimulating both proliferation and differentiation of neural stem cells, and still higher atRA concentrations inhibit cell proliferation and affect the cognitive function and behavior (Kane et al., 2010; Hu et al., 2016, 2020; Stoney, McCaffery, 2016; Stoney et al., 2016; Mishra et al., 2018). High CYP26B1 expression, apparently decreasing the atRA level, is associated with intense neurogenesis in the hippocampus of adult tame rats, earlier demonstrated by Huang et al. (2015). Our results seem to be consistent with the negative effect of atRA on neurogenesis in studies by P. McCaffery’s (Stoney et al., 2016) and Zhou’s (Hu et al., 2016, 2020) teams. It appears that CYP26B1 inhibition reduces cell proliferation in the subgranular zone of the hippocampus in mice (Stoney et al., 2016). Probably, the atRA content in the hippocampus of tame foxes is close to the maximum level enhancing neurogenesis. The lower expression of CYP26B1 in aggressive animals is associated with even higher atRA contents and, probably, lower neurogenesis. The unselected foxes, demonstrating lower neurogenesis than tame ones, seem to be in the middle between tame and aggressive. Note that genome-wide comparisons between village dogs and wolves and between humans and chimpanzees has also demonstrated differences in the atRA system (Theofanopoulou et al., 2017; Pendleton et al., 2018).

The effects of vitamin A and atRA on the HPA axis are complex and controversial. Dexamethasone upregulates the expression of the Aldh1a1 gene for an enzyme involved in atRA synthesis (Gil-Ibáñez et al., 2014). Chronic exposure to atRA and other retinoic acid forms enhances HPA axis activity and causes depression (Bremner, McCaffery, 2008; Cai et al., 2015). In particular, atRA induces overexpression of the Crf, Crfr1, and Avp genes in the hypothalamus (Cai et al., 2015). Probably, the lower HPA activity in tame foxes is partly related to the weakening of the atRA system. However, it should be noted that retinoic acid can, in contrast, lower the level of glucocorticoids, and, besides, it can exert an inverse effect on target tissues (Bonhomme et al., 2014; Hélène et al., 2016).

The detected high level of ADRA1A mRNA in the dorsal hippocampus of tame foxes is of special interest. This change may reflect the lower anxiety and elevated adult neurogenesis in the hippocampus as a result of lower HPA axis activity in the selection for tame behavior. Although the dorsal hippocampus contributes less to the regulation of stress responses and anxiety, its effects have been described in (Weaver et al., 2004; Gulyaeva, 2019). ADRA1A is presumed to play a role in the attention deficit disorder (Elia et al., 2009). The mechanisms mediating the effect of ADRA1A in behavior regulation are poorly understood, but it is known that long-term ADRA1A stimulation mitigates depression-like behavior and anxiety, and tricyclic antidepressants increase the ADRA1A receptor density in the forebrain of rodents (Deupree et al., 2007; Doze et al., 2009, 2011). Studies of the subependymal zone of lateral ventricles, which is another adult neurogenesis region along with the subgranular zone of the hippocampus, in transgenic mice demonstrate an association between high ADRA1A expression and high neurogenesis rate (Gupta et al., 2009). It is conceivable that ADRA1A also participates in hippocampal neurogenesis (Doze et al., 2011).

## Conclusion

To sum up, we analyzed separately the dorsal and ventral hippocampi of foxes selected for contrasting behaviors and revealed differential expression of the NR3C2, CYP26B1, and ADRA1A genes, associated with both hippocampal neurogenesis and HPA axis regulation. Further studies of the expression of functional groups to which these genes belong are expected to shed light on hitherto unknown molecular mechanisms of domestication in general and on stress response weakening, elevated neurogenesis, and changes of the attitude to humans in domesticated animals in particular.

## Conflict of interest

The authors declare no conflict of interest.
